# 85 Changes in Burn Surgery Operative Volume and Metrics Due to COVID-19

**DOI:** 10.1093/jbcr/irac012.088

**Published:** 2022-03-23

**Authors:** Joshua S Yoon, Kimberly H Khoo, Arya A Akhavan, Tomer Lagziel, Carrie A Cox, Julie Caffrey, Charles S Hultman

**Affiliations:** R. Adams Cowley Shock Trauma Center, Division of Plastic Reconstructive and Maxillofacial Surgery, Herndon, Virginia; Department of Plastic and Reconstructive Surgery, Johns Hopkins University School of Medicine, Baltimore, Maryland; Johns Hopkins Hospital, North York, Ontario; Johns Hopkins University School of Medicine / Sackler School of Medicine, New-York Program, Tel-Aviv University, Rockville, Maryland; Johns Hopkins Bayview Medical Center, Baltimore, Maryland; Johns Hopkins, Baltimore, Maryland; Johns Hopkins University School of Medicine, Baltimore, Maryland

## Abstract

**Introduction:**

Due to COVID-19, hospitals have had to undergo drastic changes to operating room (OR) policy to mitigate the spread of the disease. Elective surgeries were cancelled, and some ORs were repurposed to help withstand a surge of COVID-19 patients. Given these unprecedented measures, we aim to look at the changes in operative volume and metrics of the burn surgery service at our institution.

**Methods:**

An IRB-approved single-institution retrospective review was conducted by querying our institutional OR database. We obtained case lists and OR metrics for the months of March to May for 2019, 2020, and 2021, which correspond with pre-COVID, early COVID (period without elective cases), and late COVID (period with resumed elective cases). Inclusion criteria were cases related to burns. These cases were then divided into the following groups: excision only, grafting only, excision and grafting, laser scar procedures, secondary reconstruction without grafting or flaps, secondary reconstruction with grafting, and secondary reconstruction with flaps. Types of cases and operative metrics were compared amongst the three time periods.

**Results:**

The total number of cases performed by the entire hospital during 2019, 2020, and 2021 was 2375, 1184, and 2265 respectively. During those times, the burn surgery service performed 174, 124, and 212 total cases (138, 103, and 114 burn related cases) respectively. Compared to the hospital, the burn service had a smaller decrease in volume during early COVID (28.7% vs. 50.1%) and exceeded pre-pandemic volumes during late COVID (+21.8% vs. -4.6%). There was a significant increase in excision and grafting cases in early and late COVID periods (41, 84, 74 respectively; p < .0001 and p < .002). There was a significant decrease in laser scar procedures that persisted even during late COVID (69, 0, 14 respectively; p < .0001). The projected and actual lengths of cases significantly increased and persisted into late COVID (p < .01). The average length pre-COVID and late COVID were 109.9 ± 78.5 and 138.2 ± 79.2 minutes.

**Conclusions:**

COVID-19 related OR closures lead to an expected decrease in the number of overall cases and elective cases. However, there was no significant decline in the number of burn specific cases performed. The elective cases were largely replaced with excision and grafting cases and this shift has persisted even after elective cases have resumed. This change is also reflected in increased operative times.

